# Hyaluronic Acid in Topical Applications: The Various Forms and Biological Effects of a Hero Molecule in the Cosmetics Industry

**DOI:** 10.3390/biom15121656

**Published:** 2025-11-26

**Authors:** Catherine Zanchetta, Amandine Scandolera, Romain Reynaud

**Affiliations:** 1 Science and Technology, Givaudan France SAS, 31520 Ramonville Saint Agne, France; 2 Science and Technology, Givaudan France SAS, 51110 Pomacle, France

**Keywords:** human skin, hyaluronan, cosmetic, safety, penetration, aging

## Abstract

Background: Hyaluronan, or hyaluronic acid (HA), is a glycosaminoglycan with structural and signaling functions playing key roles in human skin homeostasis. It ensures hydration and biomechanical properties of this tissue as well as regulates cell adhesion, migration, proliferation, and inflammation. Its biocompatibility, viscoelastic properties, biological functions, and large-scale sustainable bioproduction made this polysaccharide a hero molecule of the cosmetic industry. Methods: A literature search was conducted to discuss the skin and hair benefits of the external use of HA and its derivatives. Four main questions were addressed: What are the different forms of HA in cosmetic formulations? What about their safety? Does HA penetrate human skin and hair? What are the benefits and mode of actions of HA, and its derivatives, in the fields of cosmetic and dermatology? Results: The analysis revealed HA below 100 kDa to penetrate skin, and lower molecular weight being able to reach the dermis. The safety of HA-containing formulations has been evaluated in several clinical trials and is supported by independent reports of commercial ingredients. We described HA molecules having beneficial effects on skin and hair, as well as their mode of action. Conclusions: This review provides comprehensive information on the nature and efficacy of topical HA, and its derivatives, in cosmetic applications, with an emphasis on hair care. New areas of research were highlighted as the vectorization of high-molecular-weight HA.

## 1. Introduction

Hyaluronan, or hyaluronic acid, herein called HA, is ubiquitous in vertebrates, also found in bacteria, and was firstly isolated in the 1930s by Meyer and Palmer in bovine vitreous humor [1], where it is highly concentrated [2]. In human skin, HA is the most abundant glycosaminoglycan and ensures an important structural and stabilizing role in the extracellular matrix, together with sulphated glycosaminoglycans and proteoglycans [3]. On the other hand, HA is a signaling molecule playing a role in wound healing, regulating inflammation, and cell proliferation [4]. HA displays a large panel of existing and prospective medical applications: treatment of osteoarthritis by intra-articular injections, eye lubricant in ophthalmology, anti-inflammatory and healing molecule in dentistry, biomaterial for tissue engineering, and as promising vehicle in cancer therapy and drug delivery [5,6,7]. In dermatology, HA has been proven to treat wounds, rosacea, or seborrheic dermatitis [8]. The polymer is used in aesthetic procedures in the form of injectable fillers and in cosmetic and nutricosmetic formulations to improve aging signs and skin moisturization [9]. This diversity of applications is made possible by the multitude of molecules derived from the native high-molecular-weight polysaccharide. Hydrolysis and functionalization of HA aim at improving its intrinsic characteristics in terms of stability, gel-forming capacity, skin penetration, and to bring new functions to the polymer [10].

Topical application of HA is a non-invasive treatment, with proven efficacy in skin care [11], but that is often overlooked in favor of aesthetic procedures. This work aims at providing an overview of HA and related molecules used in topical treatment of hair and skin, as well as in cosmetic applications. The safety and skin/hair penetration of these compounds are covered, as well as their clinical benefits and modes of action.

## 2. Methods

A literature search was achieved to answer four main questions: (1) Would topical use of HA lead to skin and hair penetration of the molecules? (2) What biological benefits are demonstrated on the skin in clinical trials? (3) What mode of action can be defined from in vitro and preclinical experiments? (4) What is the potential of HA in hair care?

The search was conducted across Pubmed, Semantic Scholar, and Google Scholar databases for English written papers in the period 1995–2025, using the combination and free text words suitable for each question in title, keywords, and abstract: “hyaluronic”, “hyaluronan”, “hyaluronate”, “skin”, “hair” “topical”, “cream”, “serum”. In order to limit our search to external use of HA, the words “oral”, “filler”, “injection”, and “injectable” were excluded. Studies referring to use of HA on skin or hair surface, were selected for cosmetic purposes. One paper reporting the use of HA in nail care was included. Studies providing a minimum description of the tested molecule (trade name and/or molecular weight at least) and conducted in human or human models were prioritized. This review does not cover the use of HA in drug delivery.

Data analysis, according to previously listed inclusion and exclusion criteria, led to the selection of 10 articles for question (1) (see Table 1), 19 articles for question (2) (see Table 2 and Table 3), 19 articles for question (3) (see Table 4), and 9 articles for question (4) listed in Section 3.3.

Along the text, low-molecular-weight HA (LMWHA) refers to molecules below 50 kDa, medium-molecular-weight HA (MMWHA) is used for polysaccharide from 100 to 500 kDa, and high-molecular HA (HMWHA) designates the ones over 700 kDA.

## 3. Biological Functions of HA in Human Skin

Our objective here is to provide concise and updated information to help the understanding of the topical HA biological effects that are developed in Section 4 and Section 5. The reader would find comprehensive information in the references cited below.

### 3.1. Structural and Physico-Chemical Properties

**Figure 1:**
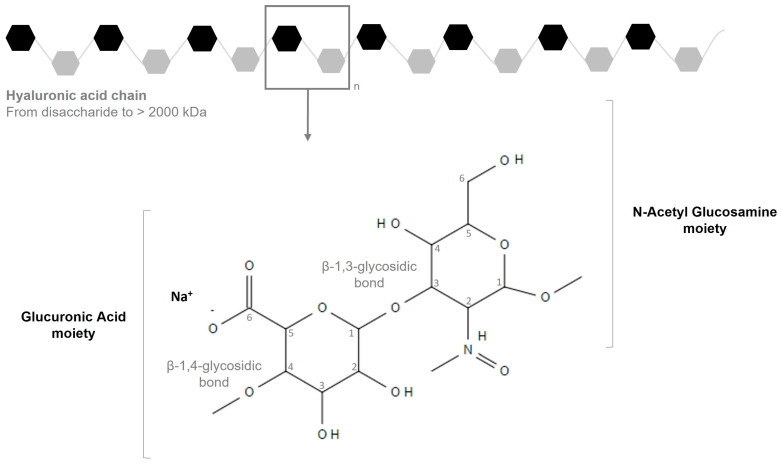
The molecular structure of sodium hyaluronate, the predominant commercial ingredient in cosmetics. Adapted from [16].

HA is a linear, non-sulphated, high-molecular-weight polysaccharide, weighing up to 6000 kDa [12]. It is made of repeating units, composed of D-glucuronic acid and *N*-acetyl glucosamine bounded by β-1,3 and β-1,4 linkages, respectively (Figure 1) [13]. At physiological pH, HA is anionic, due to the presence of negatively charged carboxyl groups. Therefore, it forms salt with cations Na^+^, K^+^, Ca^2+^, and Mg^2+^, which have elevated hydrophilicity. In aqueous solution, HA has a coil structure that is able to trap water within the helix [2]. Hydrophobic interactions and hydrogen bonding between HA chains favor the formation of a network, increasing the water retention [14]. This organization makes HA solutions have a high viscosity proportional to the concentration and molecular weight of the polymer [15].

### 3.2. Localization, Synthesis, Degradation, and Cell Surface Receptors

HA is most abundant in skin, compared to other organs, and is the major component of the extracellular matrix in which cells are embedded and connected. The extracellular matrix is more developed in the dermis than in the epidermis [17]. In the epidermal compartment, HA is mainly distributed in the extracellular space, and synthesis is higher in proliferating basal and spinous layers [17,18]. The highest amount of HA is found in the dermis, and synthesis by fibroblast is particularly high in the papillary dermis.

The half-life of HA in the skin is about one day, suggesting rapid synthesis and degradation to ensure the turnover. HA is extracellular and associated with proteoglycans, proteins; pericellular and attached to the cell membrane via the cluster of differentiation 44 (CD44) receptor; and may be intracellular. In cells, it is connected with HA-synthesizing (HAS) enzymes, HAS1, 2, 3, anchored in the membrane. Each enzyme produces a specific molecular weight and synthesized HA at the inner face, with possible translocation of the polysaccharide in the extracellular space [19]. Intracellular localization of HA was reported, and cellular HA may play a role in cell cycle, RNA translation, and splicing as well as on autophagy [20].

HA degradation is achieved by hyaluronidases (HYALs) or under oxidative conditions, forming oligomers of different sizes. HYAL1, HYAL2, CEMIP (cell migration-inducing hyaluronidase, also called HYBID or KIAA1199), and TMEM2 (transmembrane protein 2) are active in both the dermis and the epidermis [21,22]. Enzymes are extra- or intracellular, degrading HA outside cells or in endosomes or lysosomes. In the dermis, LYVE1 (lymphatic vessel endothelial hyaluronan receptor-1) is involved in HA degradation and uptake by lymphatic system for clearance through the lymph [4].

### 3.3. Roles of HA in Human Skin

Extracellular matrix is a complex, organized structure, interconnecting different elements: glycosaminoglycans as HA; proteoglycans, growth factors, and structural proteins as collagen. HA predominates in this architecture, where it plays different roles [23].

HA ensures integrity and stability of the extracellular matrix by creating a cohesive network between the different components. Extracellular HA is stabilized by interactions with proteoglycans, such as versican and aggrecan, and proteins such as collagen, leading to a supramolecular structure forming an amorphous gel surrounding cells. The ability of HA to form networks, and its viscoelastic properties, contribute to the architecture and elasticity of the tissue [4,24]. Skin hydration also participates in the biomechanical properties of the skin [25]. Thanks to its water-binding properties, HA hydrates the extracellular matrix and regulates water content in tissue, such as by interacting with aquaporin 3, responsible for transmembrane water transport [26]. Moreover, hydration promotes the circulation of ion solutes and nutrients [21].

Although HA was suggested to act as a ROS scavenger [27,28], it mainly displays biological antioxidant effect by indirectly interacting with regulators of the cellular redox status [29,30,31]. For example, HMWHA (1200–1300 kDa) decreased H_2_O_2_-induced oxidative stress in human skin fibroblasts, increasing superoxide dismutase and catalase activities, as well as reducing malondialdehyde [32].

HA would have potential antimicrobial effects on *Staphylococcus aureus* and *Escherichia coli* that could rely on the ability of the polysaccharide to interact with bacterial enzymes tyrosyl-tRNA synthetase and toposisomerase II DNA gyrase, as suggested by molecular docking investigations [28].

HA promotes biological effects through interaction with binding proteins at the cell surface, mainly CD44 but also LYVE1, RHAMM (receptor for hyaluronan-mediated motility), TLRs (toll-like receptors), and ICAM-1 (intracellular adhesion molecule). HA facilitates cell adhesion, migration, proliferation, differentiation, and communication, and also participates in the regulation of inflammation, angiogenesis, wound healing, and immunity [4].

In the epidermis, HA-CD44 interaction contributes to the polarization of keratinocytes and to the lipid synthesis in the granular layer, which are important features for the establishment of the cornified envelope of future corneocytes, and thus for barrier function [17]. An overexpression of *HAS3* was observed in the epidermis of patients with atopic dermatitis and was related to inflammatory conditions [17].

HA actively participates in all steps of the wound-healing process [33]. Injury generates LMWHA, capable of triggering an inflammatory response, notably through its interaction with CD44 and TLRs. The purinergic receptor P2X7 was also identified as a mediator of the beneficial effect of MMWHA (100 and 300 kDa) in a scratch test assay [34]. Inflammatory events induce a rapid accumulation of HMWHA at the site of injury, providing a matrix for facilitating cell migration, thus initiating tissue repair [22]. HA contributes to matrix remodeling and re-epithelialization by modulating matrix metalloproteinase, collagen synthesis, and increasing vascular endothelial growth factor [22,35]. Inflammatory response initiates the activation of local immune response to prevent microbial infection. HA of LMWHA and HMWHA also exhibits anti-inflammatory properties [22,30] and may also participate in inflammation resolution.

### 3.4. HA in Aging

HA has important roles in skin hydration, biomechanical properties, immunity, and repair. All these functions are altered by the aging process.

Intrinsic and extrinsic aging, related to environmental factors, are associated with visible signs: loss of hydration, decreased tissue elasticity, appearance of wrinkles and facial sagging, and pigmentation disorders [36]. At the cellular level, important components of the extracellular matrix (HA, collagen, elastin…) decrease along the life in association with increased DNA damage and inflammation [37]. Resulting cellular senescence prevents growth and renewal, making senescent cells accumulate in tissues. A possible link between fibroblasts senescence and HA was established by comparing the expression profile of miRNAs in dermal cells from donors of varying ages [38]. The miR-23a-3p targeted the HAS2, leading to a decrease in HA in aged fibroblasts.

Skin aging is characterized by a slowdown in HA synthase activity and an increase in hyaluronidase activity, a downregulation of CD44 and RHAMM that are involved in cell proliferation and migration, as well as a decrease in HA-binding proteins regulating cell growth and differentiation [23,33,39]. The loss of HA is most significant in the epidermis and results in low keratinocyte proliferation, thinner epidermis, and decreased skin hydration [33]. The hyalurosome is a multiproteic complex, combining CD44, HAS3, and heparin-binding epidermal growth factor and its receptor. Deficiency of the hyalurosome would be associated with premature cellular senescence [40].

Exposure to ultraviolet (UV) mainly drives extrinsic aging and causes premature skin alteration. In the papillary dermis of photoaged skin, the amount and molecular size of HA decreased, and CEMIP hyaluronidase may be responsible for excessive HA degradation [41]. Oxidative stress generated by UV exposure also contributes to the direct degradation of HA [21]. Moreover, decreased HA synthase activity (HAS1 and HAS2) was observed in the dermis of UV-exposed skin [4,39]. Shortening and low amounts of HA reduce its capacity to bind water, and form an extensive network by hydrogen bonding, affecting the moisturization and stability of the extracellular matrix. The weakening of the network was proposed to play a role in the cellular senescence and the regulation of inflammation [39].

## 4. The Different Forms of HA-Based Ingredients, Their Safety, and Interaction with Skin

Natural presence of HA in human skin makes it biocompatible. It is abundant in the cutaneous tissue, where it has wide biological functions. For these reasons, several dermatological and cosmetic applications of HA emerged to moisturize, repair, and rejuvenate skin, to care for the hair, and to treat skin disorders. In this section, we describe HA-related molecules employed in the cosmetic field. We also discuss their safety and their interaction with skin microbiota before deeply describing their ability to penetrate skin and hair.

### 4.1. The Molecules Behind the Term HA

Different terminologies are assigned to HA. The basic form is called hyaluronan and the acid one is called hyaluronic acid, abbreviated as HA in this article. Sodium hyaluronate, potassium hyaluronate, and calcium hyaluronate refer to the salts of HA. MMWHA and LMWHA are derived from the chemical or enzymatic cleavage of polymer chains [42], and thus are generally referred to as hydrolyzed HA or hydrolyzed sodium hyaluronate. The most widespread INCI (International Nomenclature for Cosmetic Ingredients) name in cosmetic products is by far sodium hyaluronate (Figure 1) (source: GNPD database Mintel Group Ltd., London, UK, https://www.mintel.com/, accessed on 25 February 2024).

Several types of HA are commercialized for cosmetic purposes. According to the United States Food and Drug Administration data from 2023, sodium hyaluronate shows the highest frequency of use, being incorporated in 4713 formulations [43,44]. This molecule is a high-molecular-weight sodium salt of HA that could be qualified as “native” as it did not undergo chemical modification after extraction from raw material. This HMWHA is also designated in its non-salt form by the term “hyaluronic acid” and is encountered in 663 formulations. This is followed by the hydrolyzed HA (476 formulations) and the derivative sodium acetylated hyaluronate (455 formulations). All other ingredients, reported in 204 compositions, may be derivatives obtained by crosslinking HA with itself or other polymers, by grafting functions or molecules onto HA, or vectorization of HA [14]. These modifications aim at improving the performance of HA, while maintaining its native properties as biocompatibility [30].

HMWHA represent the first generation of HA ingredients. In cosmetics, it is obtained from rooster combs and bacteria through the fermentation process, and has exactly the same structure independently of the origin [45,46]. These two sources were demonstrated to have low levels of impurities, and their control is of major importance in the safety of HA ingredients [47,48].

**Figure 2:**
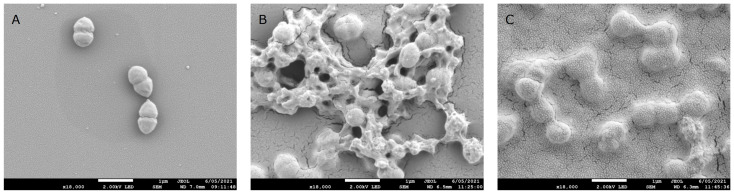
SEM pictures of *Streptococcus* sp. cells cultivated in bioreactors for the production of HA at different steps of the process. (**A**) During the growth phase, typical cells form in pairs (magnification ×18,000); (**B**) at the beginning of HA synthesis, the polymer creates a network between cells (magnification ×18,000); (**C**) the culture medium reach high viscosity due to HA increasing concentration and cells got stuck in the polymer matrix (original images from Givaudan Active Beauty Science & Technology department).

In the 1940s, HA was identified in the capsule of the *Streptococci* from group A and C [49], playing the role of virulence factor. Forty years later, the Shiseido company patented [50] and started the production of HA from *Streptococcus equi* subspecies *zooepidemicus*, which remains a predominant industrial route today [51]. Figure 2 presents SEM pictures of *Streptococcus* sp. during the production of HA in a bioreactor. The process has, nevertheless, continuously been improved by using non-pathogenic strains as *Bacillus subtilis* or by engineering non-natural producers such as the yeast *Pichia pastoris* [51]. Together with the optimization of fermentation and downstream process, these innovations improved yield, purity, and precision in the molecular weight [52,53,54].

As HA displays different biological effects according to its size, the need for low-molecular-weight compounds arose. Thus, hydrolyzed HA represents the second generation of cosmetics HA ingredients.

To enhance the intrinsic physico-chemical properties of hyaluronic acid (HA) or to impart novel functionalities, extensive efforts have focused on its chemical modification, leading to the emergence of so-called third-generation HA derivatives. Numerous modification strategies have been reported, encompassing acetylation [55], cationization [16], various cross-linking approaches (either self-cross-linking or interpolymeric cross-linking with other biopolymers) [56,57], as well as the grafting of functional molecules onto the HA backbone [58,59]. These chemical alterations enable modulation of parameters such as hydrophilicity, charge distribution, mechanical strength, and biological activity. Comprehensive review articles have systematically summarized the available modification pathways of HA, categorized according to the reactivity of its distinct functional groups [60].

### 4.2. Safety of HA in Topical Use

Due to its natural and ubiquitous presence in the human body, HA is demonstrated to be safe and biocompatible [61]. Indeed, the wide applications of the polysaccharide in medicine, aesthetic, cosmetic, and food supplement provided an extensive literature on its safety profile [62,63,64,65,66]. As we previously mentioned, several forms of HA are available on the cosmetic market and listed in the International Cosmetic Ingredient Dictionary & Handbook edited by the Personal Care Products Council. Independent authorities set the standard for cosmetic products, which include the US FDA or the European Parliament and Council Regulation [67]. In addition to regulatory bodies, the ISO provides guidelines for good manufacturing practices and quality control.

The Cosmetic Ingredient Review program provided two reference documents on the safety assessment of the most common HA commercial ingredients: HA, sodium hyaluronate, potassium hyaluronate, hydrolyzed HA, hydrolyzed sodium hyaluronate, hydrolyzed calcium hyaluronate, and sodium acetylated hyaluronate [43,44]. Based on the existing literature and unpublished data provided by industrialists, experts concluded that the seven categories of ingredients are safe in cosmetics, according to the practices and concentrations reported at the time of evaluation. They also underlined the importance of impurities in the safety of HA, which depends on the source of polysaccharide, among other parameters [47]. The safety of LMWHA (3 kDa) was recently addressed through in vitro and ex vivo experiments, and no adverse effects were observed [68]. Clinical trials presented in part 3 also provide experimental evidence that topical HA is well tolerated in formulations.

### 4.3. Interaction of HA with Skin Microbiota

Methods to evaluate the impact of cosmetic products on skin microbiota were developed in the past years, based on the profiling of cutaneous bacterial communities either by sequencing technologies [69,70,71] or through a culture-based approach [72].

Interest in the link between HA and skin microbiota is relatively new. Bacterial community changes were measured through *16S* sequencing on the face of 18–24-year-old women applying sodium hyaluronate (200–400 kDa) for 28 days [73]. The study demonstrated that HA reduced the abundance of *Cutibacterium* sp. and of the pathogenic bacteria *Streptococcus aureus*, and it increased the population of beneficial bacteria. In another study, the short-term impact of a cationic HA was evaluated by a culture-based method, indicating minimal disturbance of skin microbiota akin to physiological saline [74].

These data tend to demonstrate that HA does not adversely affect skin bacteria and could have beneficial effects that need to be explored. Interesting reports from the analysis of the gut microbiota revealed that micro-organisms metabolize HA and produce short-chain fatty acids [75]. In mice, *Bacteroides* were observed to cleave HA in oligosaccharides passing through the intestinal barrier [76]. What about the metabolism of HA at the skin surface? Does HA’s molecular weight diversely influence cutaneous micro-organisms? Does the age-related modification of skin microbiota impact HA metabolism at the skin surface? These topics remain to be explored.

### 4.4. Skin Penetration

Reaching living cells determines the capacity of ingredients to exert biological effects within the skin. While HA is strongly hydrophilic, structural data suggest that hydrogen-bonded secondary structures occur in the hydrated state, creating arrays of adjacent CH-groups with hydrophobic character, and conferring amphiphilic properties that could facilitate *stratum corneum* penetration [77]. Several methodologies and HA of different molecular weights have been used to describe the mechanisms of penetration, the quantity of HA that can reach skin compartments, and if HA can reach dermal cells.

Past and recent studies agree that the permeability of human skin to HA depends on the polymer’s size, and that molecules below 400 kDa can diffuse within the *stratum corneum* at least [78,79,80,81,82]. The polymer’s ability to reach the epidermis and the dermis was established in several studies, and is related to the formulation, concentration, number of applications, skin model, and detection method used. We reviewed here studies conducted in human skin that are summarized in Table 1.

**Table 1:** Non-exhaustive list of references that evaluated HA penetration in human skin, presented in order of publication date (most recent first).

Reference	HA MW (kDa)	HA Concentration	Dose	Formula	Method	Model	Observation
[68]	3	3%	643 µg/cm^2^	Aqueous solution	Raman spectroscopy	Skin explants	HA penetrates up to 60 µm
[78]	400	3–13.4 mg.g^−1^	56.3 mg/1.8 cm^2^	Water, benzyl alcohol 0.5% (*v*/*v*)	HA Radiolabeling, biopsy, imaging	In vivo, forearm	HA is absorbed up to the dermis
[83]	200–325	1 mg.mL^−1^	200 µL/1.77 cm^2^	Phosphate buffer saline solution (50 mM, pH 4)	Franz diffusion cell	Full-thickness abdominal skin	HA penetrates up to the dermis but only 2–3% of the applied dose enters the skin
[79]	6.5 185	2%	-	Aqueous solution	Franz diffusion cell Confocal microscopy of fluorescein-labelled HA	*Stratum corneum* with epidermis isolated from abdominal skin	Fluorescein-labelled HA permeate the *stratum corneum* and epidermis, depending on MW
[80]	1000–1400 100–300 20–50	1%	300 µL/0.79 cm^2^	Aqueous solution	Raman spectroscopy	Dermatomed skin from abdominal skin	HA 1000–1400 kDa did not permeate skin, HA 20–300 kDa fully permeates the *stratum corneum*, and 20–50 kDa HA reaches the deeper epidermis
[84]	2.044	0.34%		Serum	Quantification of HA released in culture medium	Epidermal barrier model	HA crossed the multilayer of keratinocytes
[85]	10	-	2 mg/cm^2^	Cosmetic formula	Matrix-assisted laser desorption/ionization mass spectrometry imaging	Skin explants from abdominoplasty	HA was detected in each skin layer
[86]	2 1000	0.1%	-	-	Franz type diffusion cell, Fluorescein labelling, Fluorescence microscopy	Dermatomed abdominal skin in Franz-type cell chamber	2 kDa fluorescein-labelled HA penetrated up to the dermis while 1000 kDa HA did not pass through the *stratum corneum*
[82]	1000–1400 20–50	0.5% 100–1400 kDa–1% 20–50 kDa	About 112 mg HA in 56 cm^2^	Emulsion	Tape stripping and quantification by Elisa assay	Clinical study, forearm	HA diffuse and accumulate in the *stratum corneum* following repeated application
[81]	10 400–1000	2 mg.mL^−1^	200 µL	Aqueous solution	Rhenium-tricarbonyl labeling, infrared microscopy, and fluorescence imaging	Skin biopsies in Franz cell chamber	After 24 h, the rhenium-tricarbonyl-labelled 10 kDa HA is homogeneously distributed in the *stratum corneum* with very weak amount detected in the viable epidermis

In 1999, Brown et al. demonstrated that a 400 kDa HA is needed to penetrate the skin in vivo [78]. The penetration of such high-molecular-weight compounds was rationalized by the ability of the polymer to adopt flexible and dense conformation [79].

The use of Raman spectroscopy allowed non-invasive observation of HA penetration in the skin [80]. Three HA were evaluated on dermatomed skin samples (abdomen) at 1% in water: 20–50 kDa, 100–300 kDa and 1000–1400 kDa. For all of these HA, the strongest signal was recorded in the *stratum corneum*. The 20–50 kDa HA was present in the deepest epidermis layers (up to 100 µm), the 100–300 kDa HA was present in the superficial layer of the epidermis under the *stratum corneum* (up to 50 µm), while the 1000–1400 kDa HA was found only in the *stratum corneum* (up to 25 µm).

A 10 kDa HA was labelled with rhenium-tricarbonyl, applied on human skin biopsies and observed by fluorescence and infrared detection. The labelled HA homogeneously penetrated the *stratum corneum* 24 h after application, but was slightly distributed in the viable epidermis [81]. Using matrix-assisted laser desorption/ionization mass spectrometry imaging, Legouffe and collaborators established the penetration profile of a similar-molecular-weight HA (10 kDa) formulated in a cosmetic chassis and applied for 6 days on skin explants [85]. Applying the formula increased HA level in the epidermal and dermal compartments. This suggests that either repeated application or a longer treatment duration resulted in higher HA concentration in the dermis. The content of HA in the skin following external application was determined by Grégoire and collaborators [82]. The authors developed a quantitative methodology combining tape stripping and ELISA detection. A combination of 100–1400 kDa and 20–50 kDa was formulated in emulsion and applied on the forearm of participants, close to “real-life” cosmetic use. The background signal of HA synthesized within the skin prevented a significant measure in the epidermis and in the dermis. The clinical study confirmed HA accumulates and is maintained in the *stratum corneum* after 7 days of application, from below 10 ng/cm^2^ on day 0 to more than 1000 ng/cm^2^ on day 7.

Smaller molecules (<10 kDa) have been developed in the past, particularly to improve skin penetration. HA of 2 kDa were observed to cross the epidermis in a cellular model of the epidermal barrier [84] and to reach the dermis of excised human skin [86]. The 2 kDa was distributed heterogeneously in the form of aggregates within extracellular space of keratinocytes, while it had uniform distribution in the fibroblasts and surrounding cells [84]. A 3 kDa LMWHA was found to reach 60 µm beneath the skin surface after 8 h treatment [68]. According to the authors, this penetration was lower than previous observations on 50 kDa HA and they suggested that the delay could result from a transcellular route of the 3 kDa HA.

The kinetic of penetration of twelve HA ranging from 0.4 kDa to 2000 kDa, including a cross polymer, was recently realized on porcine skin [68]. As previously described, authors established a negative correlation between the amount of material penetrating the skin and the molecular weight. Their analysis demonstrated that 60% of the penetration variability was explained by the molecular weight. Except cross polymer, 27–39% of the material deposited on the *stratum corneum* penetrated the epidermis for all molecular weights tested, starting after 30 min and reaching a plateau after 4 h. More than 50% of the applied material reached the dermis for molecules from 0.4 to 100 kDa, and the highest value recorded was 75% for the 0.8 kDa. About 25% of 200 and 500 kDa HA reached the dermis. The HA content was stable in the dermis after 8 h. Molecules were not retrieved in the collecting chamber placed below the dermis, suggesting that they were retained within the tissue.

The mechanisms by which HA penetrates the skin are poorly understood and were mainly studied in animal models. Brown et al. hypothesized that topical HA penetration could be mediated by extracellular diffusion and/or active transport [78]. They observed that some dermal cells internalized the labelled polysaccharide, which seemed to interact with components of the *stratum corneum*. Later research confirmed HA (190–200 kDa) to have a great affinity for keratins and suggested it crosses the *stratum corneum* and the epidermis through corneocytes [79]. Nevertheless, an intercellular route through the epidermis was observed for a fluorescein-labelled 10 kDa HA in reverse micelle formulation [87]. Witting and collaborators established that HA induces a conformation change of the keratin, promoting the conversion of α-helical structures in β-sheets, thus increasing *stratum corneum* permeability [79,88]. LMWHA of 5 kDa impacted the keratin structure more than a 100 kDa one [88]. Skin hydration, which increased in relation to decreasing molecular weight of HA, was also hypothesized to improve molecule transport within skin and explained the retention of HA within the epidermis. Hair follicles also constitute a penetration pathway for topical treatment—especially in the scalp—as their structure invaginates deeply into the dermis [89]. In conclusion, affinity of HA for keratin may help the polysaccharide in the penetration of *stratum corneum*, especially low-molecular-weight compounds, and both intracellular and extracellular routes may exist. It has to be defined which pathway predominates and how HA’s molecular weight can affect its trajectory within skin tissue.

## 5. Beneficial Effects of Topical HA in Skin: Clinical Efficacy and Mechanisms

In the previous section, the HA skin permeation was addressed. In this section, we focused on the clinical outcomes and biological effects provided by the topical application of HA.

### 5.1. Native and Hydrolysed HA

**Figure 3:**
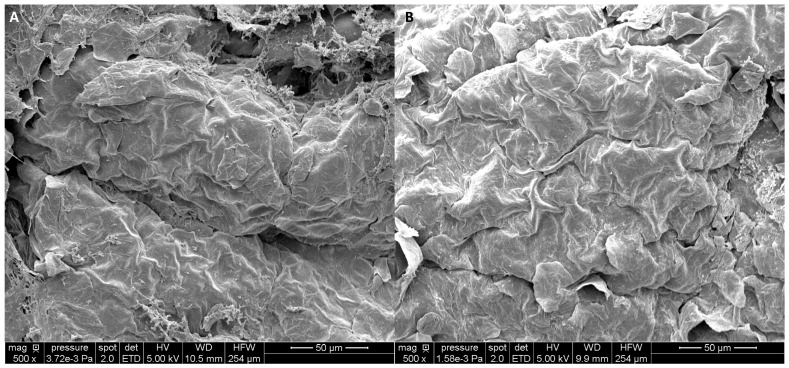
Scanning electron microscopy of (**A**) skin explants untreated and (**B**) treated with high-molecular-weight HA (1000–1400 kDa). The polysaccharide forms a film at the skin surface and increases skin smoothness. Magnitude ×500. From ref. [90].

HMWHA (>1000 kDa) are used as a viscosity modifier, film former (Figure 3), and moisturizer [61]. Rheological properties of HA depend on its molecular weight and concentration. The influence of these parameters was recently refined, and strong network formation is obtained with HA from 400 kDa [15]. In aqueous solution, hydrophobic interactions and hydrogen bonding promote aggregation of HA chains into an extended network-forming gel [14].

As previously stated, HMWHA poorly penetrates the skin, but the polysaccharide network forms a film at the skin surface that helps decrease transepidermal water loss and provides hydration [80,91]. Above its film-forming properties, HA carries a high amount of water, enhancing water retention in tissue [92]. In diluted aqueous solution, sodium hyaluronate has semi-rigid coil structure and could organize in a helix that attracts a great quantity of water [93]. Moreover, formation of intermolecular bonds between chains to form gels also increases water retention in HA. The hydration level of HA was reported to be 0.7 to 2 g of water per g of polysaccharide [94,95,96]. HMWHA (1200–2000 kDa, 0.1 to 0.5%) provides significant moisturizing effect in vivo in 28 to 60 days and even from 1h after application [91,97,98]. A better hydration of skin can explain the decrease in fine lines and better elasticity provided by HMWHA [25]. Nevertheless, in all comparative studies, their efficacy was lower than HA below 1000 kDa. In aging, dermal HA content decreases, resulting in loss of skin moisture and loosened extracellular matrix [39]. Because hydrolyzed HAs penetrate the skin, it is expected that they will bring water deeper into the tissue, improving skin plumping, and that they could interact with living cells. But as previously mentioned, LMWHA does not always penetrate deeper into the skin and its action may rely on its ability to penetrate cells [68]. Molecules below 300 kDa, and even more below 50 kDa, display several anti-aging properties: decrease in wrinkles and roughness, increase in skin elasticity and firmness, and improvement of dermal collagen score (Table 2 and Table 3).

**Table 2:** Non-exhaustive list of clinical trials evaluating the cosmetic and dermatological applications of topical HA on human skin. Studies are classified by application and chronological order (most recent first).

Application	Reference	HA (Type, Trade Name, Molecular Weight)	Study Parameters (Cohort Size, Age Range, Body Site, Placebo Availability, Formula)	Findings
Anti-Aging	[99]	Hydrolyzed sodium hyaluronate HAbooster™ 2 kDa	*n* = 21 (41–60 years), face, placebo controlled, HA 0.1% in lotion	Reduction in facial pigmented spots, eyebag sagging, and pore volume.
	[86]	Hydrolyzed sodium hyaluronate HAbooster™ 2 kDa	*n* = 8 (26–39 years), forearm and face, placebo controlled, HA 0.1% in lotion	Increase in dermal collagen score, skin elasticity, and water content. Reduction of sagging and crow’s feet/nasolabial wrinkle depth.
	[100]	Sodium hyaluronate PrincipHyal^®^	*n* = 75 (average age 55.8 years), face, placebo controlled, HA 0.5% in cream	Formula well tolerated. Increase in moisturization, elasticity, firmness, and smoothness. Decrease in wrinkle depth.
	[101]	Sodium hyaluronate in novel conformation 1200 kDA	*n* = 22 (average age 52.2 years), face, HA 0.5% in aqueous solution	Increase in skin hydration, elasticity, and density. Reduction in transepidermal water loss, roughness in the eye contour, and facial sagging.
	[97]	Sodium hyaluronate 50, 130, 300, 800, 2000 kDa (comparison)	*n* = 76 (30–60 years), face, placebo controlled, HA 0.1% in cream	All HA increased skin moisture and elasticity. Wrinkle depth was significantly reduced by 130 and 50 kDa.
Moisturization	[91]	HA 5, 8, 42, 360, 920, 1770 kDa (comparison)	*n* = 3 (20–25 years), arm, HA 0.1% in solution	Hydration level increase with decreasing molecular weight.
Dry skin (xerosis)	[98]	7 kDa 1800 kDa (comparison)	*n* = 36 (60–80 years), leg, 0.1% 7 kDa HA or 0.1% 1800 kDa in lotion, placebo controlled	Both HA increased skin capacitance with a significantly better effect of the 7 kDa compared to the 1800 kDa. No modification of transepidermal water loss or specified symptom sum score.
Seborrheic dermatitis	[102]	LMWHA Bionect Hydrogel	*n* = 13 (18–75 years), face, HA 0.2% in gel	Excellent tolerability. Reduction in erythema and pruritus.
Rosacea	[103]	LMWHA Bionect Hydrogel	*n* = 14 (18–75 years), face, HA 0.2% in gel	Excellent tolerability. Reduction in papules, erythema and dryness.

**Table 3:** Non-exhaustive list of clinical trials evaluating the cosmetic and dermatological applications of topical HA formulated with other active ingredients on human skin. Studies are classified by application and chronological order (most recent first).

Application	Reference	HA (Type, Trade Name, Molecular Weight)	Study Parameters (Cohort Size, Age Range, Body Site, Placebo Availability, Formula)	Findings
Anti-Aging	[104]	Jalubalance^®^	*n* = 91 (average age 43 years), face, HA in cream	Reduction in wrinkles and hyperpigmentation. Improvement in skin elasticity and uniformity.
	[105]	Sodium hyaluronate	*n* = 44 (average age 48 years), face, HA in formula with niacinamide *n* = 30 (35–55 years old), arm, HA in formula with niacinamide, biopsy	Improvement of fine lines, wrinkles, luminosity, smoothness, homogeneity, and plumpness. Biopsies revealed 101 mRNAs and 13 miRNAs differentially expressed compared to untreated skin. Senescence-associated secretory phenotype genes were down-regulated.
	[106]	Hydrolyzed HA Primalhyal™ 50 20–50 kDa Vectorized HA Hyalusphere™ PF 1000–1400 kDa	HA 0.5% in formula	Formula well tolerated. Wrinkle depth and length in the periorbital area were significantly reduced.
	[107]	Sodium hyaluronate Sodium Hyaluronate crosspolymer	*n* = 46 (average age 43.6 years), face, HA in serum	Increase in skin hydration. Decrease in wrinkles and erythema. Biopsies showed CD44 increase and improvement of solar elastosis.
	[108]	Hydrolyzed HA Sodium hyaluronate	*n* = 40 (30–65 years), face, HA in serum	Formula had excellent tolerability confirmed by no increase in IL-1a. Improvement in smoothness, plumping, and hydration. Decrease in fine lines and wrinkles.
	[109]	Nano HA Hyalogy^®^ Skincare	*n* = 33 (average age 45.2 years), face, HA formulated in cream, serum, and lotion	Increase in skin moisture and skin elasticity.
	[110]	Sodium hyaluronate cross polymer, and blend of 6 HA/sodium hyaluronate/hydrolyzed sodium hyaluronate Fillerina^®^ 1,5, 50, 200, 2000 kDa (mix)	*n* = 40 (25–55 years), face, placebo controlled, HA incorporated in 5 formula	Lip/cheekbone volume increase, decrease in skin sagging in face/cheekbone contour, decrease in wrinkle volume and depth.
Dermatoses (rosacea, sensitive skin, reactive skin)	[111,112]	Sodium hyaluronate	*n* = 510 (18–88 years), face, HA in formula (M89 Vichy)	The formula decreased the symptom scores of dryness, burning sensation, itching, and stinging/tingling. The prevalence of subjects with severe clinical signs (erythema, desquamation, irritation) decreased.
Dry skin related dermatoses	[113]	Sodium hyaluronate	*n* = 47 (average age 40.9 years), face, HA in formula (M89 Vichy)	The formula decreased the sensation of burning, itching, and stinging/tingling and improved skin dryness in dermatoses related to compromised barriers.

The biological mechanisms underlying such visible effects are not fully understood, but in vitro and ex vivo experiments provide insights into the possible mode of actions of HA (Table 4). Different biological models are described in Table 4—in vitro, ex vivo, and in vivo—each offer specific advantages for studying the biological effects of hyaluronic acid (HA), but they also present inherent limitations and potential biases. In vitro models allow precise control of experimental variables and mechanistic insight but often oversimplify the biological environment, lacking factors such as vascularization or immune response. Ex vivo models better preserve tissue architecture but remain limited in duration and systemic integration. In vivo models provide the most physiologically relevant context but may be influenced by interspecies differences, ethical constraints, and biological variability. Therefore, the interpretation of HA’s biological effects requires a critical perspective that integrates data from these complementary models. Considering all results together provides a more comprehensive and reliable understanding of HA’s performance and mechanisms of action.

The biomechanical properties of the skin depend on dermal extracellular matrix homeostasis, where HA is the most abundant component and decreases with skin aging. Recently, the overproduction of HA in the skin was directly correlated with wrinkle decrease [114]. To explain the clinical improvement of skin firmness, elasticity, and wrinkle reduction, it is interesting to explore how HA impacts fibroblast and dermis metabolism. A proteomic study on human dermal fibroblasts treated with 20–50 kDa HA revealed major changes in several pathways: proliferation and growth, extracellular matrix reorganization, proteoglycans and collagen biosynthesis, mitochondrial activity, cell adhesion, wound healing, immune response, and inflammation [115,116]. Several other reports confirm HA to modify the extracellular matrix by increasing collagen I and modulating matrix metalloproteinase [86,117,118]. High LMWHA (<1kDa) more efficiently promoted type I collagen without impacting matrix metalloproteinases [118]. In addition, dermal collagen scores were increased in vivo by 2 kDa HA [86].

**Table 4:** Non-exhaustive list of studies decrypting the biological effect and mode of action of exogenous HA application.

Biological Effect	Ref.	Model	HA Characteristics	Findings
Fibroblasts Extracellular matrix	[86]	Full-thickness 3D skin model	2 kDa	Promotion of collagen-type 1A1 and matrix metalloproteinase 1.
	[119]	Human dermal fibroblasts	10 kDa 1600 kDa	HA decreased total protein synthesis; did not modify collagen or HA content.
	[117]	Human dermal fibroblasts	30 kDa	Reduction in matrix metalloproteinase 1 expression.
	[115,116]	Normal human dermal fibroblasts	20–50 kDa	HA modified sphingolipids composition. Several pathways were impacted by HA: proliferation and growth, extracellular matrix reorganization, proteoglycans biosynthesis, mitochondrial activity, cell adhesion, wound healing, immune response.
	[120]	Human dermal fibroblasts (2D, 3D)	900 kDa 2300 kDa	900 kDa > 2300 kDa HA in stimulating fibroblast proliferation.
	[118]	Human dermal fibroblasts	1700 kDa HA12 fragment of 12 saccharide units HA 880 fragment of 880 saccharide units	Increase in matrix metalloproteinase 1 and 3 gene expression. HA12 enhanced collagen 1 expression and transforming growth factor-β1. HA12 and 1700 kDa stimulated type III collagen and transforming growth factor-β 3.
	[121]	Normal human dermal fibroblasts (monolayer and in 3D collagen gel)	750 kDa 2700 kDa	Independently of the molecular weight, HA modified actin cytoskeleton organization.
Epidermal-dermal junction, epidermal development	[122]	Reconstructed epidermis	50 kDa 800 kDa (topical)	50 kDa > 800 kDa in up-regulating the expression of several genes involved in Epidermal-dermal junction (tight junction, claudin, ZO1, ZO2, cadherin…), epidermal differentiation (kallikrein, repetin, keratin), and delaying aging (sirtuin).
Barrier function	[123]	Keratinocytes (HaCaT)	<100 kDa >500 kDa	Increase in the protein expression of claudin-3, claudin-4 involved in tight junction formation, and of JAM-A, a junctional adhesion molecule.
Epidermal homeostasis	[124]	Epidermis 3D model	LMWHA (topical)	Up-regulation of pro-filaggrin gene expression; increase in filaggrin protein content and caspase 14 activity, suggesting improvement in hydration through natural moisture factor enhancement.
Epidermal differentiation	[125]	Normal human epidermal keratinocytes	0.776 kDa >1200 kDa	Increase in differentiation markers gene and protein expression by both HA. HA4 increased CD44-phosphorylated protein and intracellular calcium concentration.
Epidermal differentiation	[126]	Mouse Human	Mix of 1 to 50 kDa (topical)	HA induced keratinocyte proliferation involving CD44 and induced hyperplasia in atrophic human skin. Increase in HA synthase and hyaluronidase expression not mediated by CD44.
Epidermal differentiation	[127]	Skin equivalent model	oligosaccharide	Epidermis thickening and promotion of epidermal differentiation.
Inflammation	[99]	Normal human epidermal keratinocytes	2 kDa	Reduction in H_2_O_2_-induced gene expression of TNF-α and IL1-β and improvement in cell viability. Autophagy and cell renewal markers increased secretion.
Inflammation and barrier function in rosacea	[128]	Normal human keratinocytes and BALB/c mouse model treated with rosacea inducer LL-37	0.776 kDa	Decrease in LL-37-induced pro-inflammatory cytokines in keratinocytes. In mice, decrease in inflammatory cell infiltration, interleukin 17A, and kallikrein 5, as well as increase in CD44 and filaggrin expression.
UV response	[129]	Keratinocytes (HaCat cells)	970 kDa	Suppression of UVB-induced pro-inflammatory cytokines and cell viability maintenance
	[130]	Keratinocytes (HaCat cells, normal human epidermal keratinocytes)	0.8 kDa 1200 kDa	0.8 kDa HA decreased UVB-induced interleukin 6 secretion by binding the receptor TLR4. The 1200 kDa also decreased the interleukin 6.
	[131]	Skin explants	HA in formula	Prevent the decrease in expression of skin barrier markers and the induction of inflammasome.

HA was also observed to act on the epidermal compartment at the clinical level, improving the symptoms of barrier-related dermatoses. In reconstructed epidermis, 50 kDa HA up-regulated the expression of several genes involved in epidermal-dermal junction and epidermal differentiation [122]. Up-regulation of keratinocyte differentiation markers was reported in different models (normal human epidermal keratinocytes, skin equivalent, mouse and human skin) and led to epidermal thickening [122,125,126,127]. HA also increased keratinocytes proliferation [126]. Although a body of evidence tends to suggest that HA synthesis inhibition does not impact proliferation and differentiation [132], recent results confirmed that increasing HA production accelerates keratinocyte proliferation and differentiation [133]. Moreover, HA was also observed to increase tight junction proteins in keratinocytes [123]. In an epidermal model, LMWHA increased the content of filaggrin and involucrin, as well as the activity of proteolytic enzymes degrading the filaggrin [124]. Involucrin and the products of filaggrin degradation participate in the natural moisturizing factors that help maintain water in corneocytes. Authors hypothesized that HA could promote skin hydration through the improvement in *stratum corneum* barrier function. Furthermore, they observed an increase in HA content within the basal layer of the epidermis, which could also contribute to the hydrating properties of LMWHA. Taken together, these findings suggest HA contributes to the barrier function of the epidermis. In aging, disturbance of the epidermal barrier affects skin hydration, immunity, and inflammation regulation [134]. External factors, such as UV exposure disturb this function [135]. In UV-treated skin explants, a cosmetic formula containing HA maintained the expression of involucrin [131].

Erythema characterized the visible color change related to inflammatory state in the skin. HA decreased this symptom in sensitive and reactive skin, rosacea, and seborrheic dermatitis [102,103,111,112,113]. In normal human keratinocytes, 0.8–2 kDa HA reduced pro-inflammatory cytokines induced by H_2_O_2_, or in a rosacea model [99,128]. HA alleviated the release of UVB-induced pro-inflammatory cytokines in keratinocytes [129,130]. In UV-treated skin explants, a cosmetic formula containing HA prevented the induction of inflammasome [131]. Nevertheless, depending on the biological context and its molecular weight, HA displays anti- or pro-inflammatory activities [122,136,137,138].

This duality is of great importance in the wound healing process: rupture in tissue integrity induces HA fragments to elicit the immune system through an inflammatory cascade regulated by HMWHA [30]. HA playing a role in cell differentiation and proliferation (Table 3) is used in several wound-healing applications [139].

Several limitations regarding the evaluation of HA were observed: sample size, low level of information characterizing the nature of HA, inclusion of other bioactive compounds in formulations, results obtained on limited populations that are difficult to generalize. Understanding the effects of HA in topical applications remains an open topic.

### 5.2. Functionalized HA

Functionalization of HA aims at enhancing or providing new properties to the polysaccharide. It can be considered that hydrolysis was the first way to modify HA, notably to increase its skin permeability. Here, we considered that functionalization was the chemical binding of HA with compounds or the establishment of linkages between polysaccharide chains.

Acetylated HA is the most common chemical derivative commercialized in cosmetics [44]. Acetylation improves the skin bioavailability of HA. Molecules can vary in the molecular weight of the HA and the degree of acetylation. A 15 kDA sodium acetylated hyaluronate permeated human skin explants up to 100 µm, while its non-modified equivalent was mainly detected in the *stratum corneum*, and penetrated up to 50 µm [55]. On top of that, the derivative was also less susceptible to the degradation by hyaluronidase, suggesting a higher stability of the molecule within the skin. The biological evaluation of 15 and 30 kDa acetylated HA underlined their anti-aging properties. The 30 kDa acetylated HA enhanced type I collagen secretion in normal human dermal fibroblasts [117]. For its part, the 15 kDa limited collagen degradation: it down-regulated the gene expression and protein synthesis of collagen-degrading enzymes (matrix metalloproteinases 1, 3, 9) and prevented collagen degradation in vitro [55]. The protection of collagen was related to the decrease in number of wrinkles and improved skin texture observed in vivo. Acetylation of a 90 kDa HA was suggested to improve the radical scavenging and anti-inflammatory activity properties of the polysaccharide, so that other benefits of acetylated HA could emerge in the future [140].

As previously mentioned, interaction of polysaccharidic chains in solution causes HA to form gels, but they have low mechanical stability [60]. Crosslinking of HA provides hydrogels with higher mechanical resistance, with longer stability in tissues, suitable for volume correction and tissue augmentation fillers. Nevertheless, crosslinked HA can form a cohesive film at the skin surface and was also evaluated clinically for topical application [56,141]. In human skin explants, crosslinked sodium hyaluronate induced the increase of extracellular matrix amorphous components and the reorganization of collagen fibrils, suggesting a better hydration of the matrix and a younger phenotype of the skin [142]. Collagen reorganization consisted of an enlargement of collagen fibers, smaller interfibrillar distance, and an increase in collagen bundle tortuosity. These features were characteristic of a young skin phenotype and could be explained by the hydration of collagen fibers by HA. Comparison with non-modified LMWHA and HMWHA revealed crosslinked HA to more effectively increase epidermal water content and to reduce transepidermal water loss [143]. Crosslinked HA improved skin micro-relief, probably in relation with its film-forming properties. In a clinical evaluation the molecule was confirmed to improve skin surface topography and hydration [56]. Another crosslinked HA formulated with actives in hydrogel-based moisturizer had proven efficacy against xerosis [141]. Hydrogels are a trendy topic, with great potential in cosmetics for their improved hydration and film-forming capacities [144]. The multiple ways of assembling HA chains, or crosslinking with other polymers, enable the development of a large panel of new molecules [14].

At physiological pH, the carboxyl group of glucuronic acid moieties are negatively charged, which also applies for HA. The surface of the skin also carries negative charge, limiting the interaction of HA with the tissue. Positively charged emulsions display better skin penetration [145]. Moreover, the high hydrophilicity of HA makes it less compatible with rinse-off applications. In 2023, only 8.4% of HA-containing products sold on the US market were formulated for this usage. A cationic HA was demonstrated to better adhere to the skin surface compared to its native equivalent of similar molecular weight [146]. In vivo, the cationic HA formulated in rinse-off shower gel increased the skin hydration and induced a slight overexpression of filaggrin and aquaporin-3 in ex vivo experiments [16]. Cationic HA also found application in nail care [147]. Associated with *Pistacia lentiscus* gum, the positively charged HA was selected to deeply moisturize nails and cuticles. A clinical study revealed the formulation to have improved nail appearance by reducing nail plate roughness and increasing tegument resistance.

Several other functionalized HA were evaluated for application on the skin surface. Retinoic acid-grafted HA had long-term stability and resistance to photodegradation [59]. It penetrated across the *stratum corneum* and exhibited anti-inflammatory effects. The molecule up-regulated the retinoid pathway in fibroblasts [148]. Glutamic acid-grafted HA also displayed anti-inflammatory activity in fibroblasts [149]. Chemical bonding of titanium dioxide particles to HA, for the development of sunscreen, showed promising results [58].

### 5.3. Enhanced Delivery and Biological Benefits of HMWHA

HMWHA exerts distinct biological benefits compared to its lower-molecular-weight counterparts. Due to its high chain length and extensive hydration capacity, HMWHA forms a viscoelastic film on the skin surface that reinforces barrier function, reduces transepidermal water loss, and provides a smoothing and protective effect. However, its size typically prevents effective diffusion through the *stratum corneum*, thus limiting deeper biological activity. Indeed, in vitro investigations suggested these molecules could increase collagen synthesis, remodel cell cytoskeleton, and have anti-inflammatory properties [118,121,129].

Recent technological approaches have aimed to enhance the delivery of HMWHA without chemical modification. Considering that HA conformation depends on the ionic strength of its environment, magnesium chloride has been identified among metal ions as capable of promoting the penetration of sodium hyaluronate (1100–1600 kDa) within the *stratum corneum* [150]. The divalent magnesium ions interact with the carboxylate groups of HA, reducing electrostatic repulsion and inducing a more compact polymer configuration, which favors transient diffusion into superficial skin layers. MgCl_2_-HA reached the middle layer of the *stratum corneum* via the intercellular route, not modifying the transepidermal water loss, thus suggesting it did not alter the barrier function.

Nanoparticles composed of HA and poly-L-lysine have also improved the penetration of 1200 kDa HA in the *stratum corneum* of mice [151]. These findings suggest that native HA and HA-based nanoparticles may follow distinct penetration pathways, possibly involving intercellular diffusion or follicular routes. Similarly, adsorption of a 1000 kDa HA onto activated clay facilitated entry up to 70 µm beneath the *stratum corneum*—within the viable epidermis [90]. The clay-based vectorization modified the zeta potential of HA, increasing its negative charge and sensitivity to local pH variations, thereby promoting electrostatic interactions with the skin surface. Vectorization allowed HA to better improve skin suppleness in a more prolonged time, compared to the native HA form.

A liposomal formulation was also developed to enhance the beneficial effects of HMWHA on photodamaged skin. The liposomes, produced through combined techniques of reverse-phase evaporation, high-speed homogenization, and micro-jet high-pressure processing, enabled the encapsulation of HA (1000–1500 kDa) and improved its accumulation within the epidermal layers [152]. The liposomal preparation inhibited photo-induced aging in vitro and had anti-inflammatory and wound-healing properties in a murine model of acute skin injury.

## 6. HA in Hair Care

Although HA is mainly known by consumers in facial products, its appeal in the hair care category is not new. As early as 1994, its combination with cationic cellulose polymer was demonstrated to increase the substantivity of HA on keratin after water rinsing, in order to prolong its hydration benefits [153]. Associated with collagen and chitosan, it was evaluated for hair conditioning [154]. In addition, this market is expanding: in 2005, the use of sodium hyaluronate in non-coloring hair preparations was reported in 12 formulations in the US market, while it was mentioned in 83 in 2023 [44]. However, it was not until recently that the properties and mechanism of action of HA on hair fiber were demonstrated and explained.

The hair shaft is constituted by an external envelope, the cuticle, and a central part, the cortex, both composed of the fibrous protein keratin, ensuring protection and resistance. Keratin chains in a α-helix conformation are coiled by hydrogen bounds. This tertiary structure favors the formation of disulfide bonds between sulfur-containing cysteine amino-acids, crosslinking adjacent chains, that are responsible for hair curling. Conversely, in the β-sheet conformation, disulfide bonds are less frequent and hair shaft is smoother [155]. Mechanical hair straightening causes the interconversion of keratin from α-helix to β-sheet.

A hydrolyzed sodium hyaluronate formulated in shampoo demonstrated anti-frizz effect [156]. To evaluate the impact of this HA on hair structure, confocal Raman spectroscopy was applied on fibers treated with placebo or HA-containing shampoo. As previously described in skin [88], this hydrolyzed sodium hyaluronate modified the tertiary structure of hair keratin, explaining the smoothing effect of the hydrolyzed HA: it decreased α-helix conformation, promoting the β-sheet one, and treated hair also exhibited a lower intensity of covalent disulfide bond of cysteine.

More recently, the comparison of three HA (1400 kDa, 370 kDa, 42 kDa) revealed that the 42 kDa one improved resistance and elasticity of damaged hair [157]. The fluorescent labelled LMWHA penetrated up to the cortex. Authors suggested that it may increase water-binding capacity of hair and promote hydrogen bonds within keratin chains.

In another study, HMWHA (1000–1400 kDa) and LMWHA (20–50 kDa) were formulated alone or blended in shampoo to treat (washing and rinsing) natural undyed hair locks. Both HA were detected in the cuticle, while only the blend of both sizes significantly penetrated the cortex [158]. The combination displayed straightening properties, increased hair water content, and improved the elasticity and resistance of bleached hair.

The activity of HA was not limited to structural effects. Zerbinati and collaborators evaluated a non-crosslinked HA filler formulated with amino-acids in dermal papilla cells [159]. Ingredients supported the proliferation and growth of keratinocytes and had a role in the induction of hair follicles and hair growth. It would be of great interest to compare diverse HA molecular weights in this model and evaluate their penetration in the scalp. Indeed, hair follicles are invaginations extending into the dermis, which are penetration pathways for topically applied molecules [89].

Although these data demonstrate that HA, especially below 50 kDa, can penetrate up to the hair cortex and interact with keratin, the precise mechanisms by which the polymer enter the fibers is not clear. The cell membrane complex ensures cohesion between hair cells. The intercellular transport of substances through this cement is considered as the main pathway of penetration for organic compounds, while a transcellular route exists but is less probable [160].

## 7. Conclusions and Perspectives

This review provides comprehensive information on the nature and efficacy of HA and its derivatives for topical application, mainly in cosmetics, but also in dermatological applications. A body of evidence indicate that HA is capable of reaching living cells of the epidermis and of the dermis, depending on its molecular weight. HA is clinically proven to have anti-aging, moisturizing, anti-inflammatory, and healing properties. We reviewed new area of research by discussing the use of HA in hair care and the vectorization of HMWHA.

Future investigations should prioritize foundational research aimed at elucidating the precise relationships between HA molecular size, three-dimensional conformation, and its localization within distinct cutaneous compartments. Particular emphasis should be placed on the identification of novel HA-binding receptors and the characterization of the downstream signaling cascades they initiate, as well as on the detailed mechanisms governing HA penetration, diffusion, and transcutaneous transport. While the effects of aging on HA biosynthesis and catabolism within the skin are increasingly well characterized, complementary studies are warranted to explore the dynamics of HA transport in aged tissue and its potential involvement in intercellular communication processes. These insights will be critical to guiding the rational design of biomimetic HA derivatives, leveraging advancements in biotechnology to engineer structurally and functionally optimized molecules. Such tailored derivatives have the potential to simultaneously replicate the extracellular matrix-supportive role of native HA and modulate signaling pathways relevant to cutaneous aging, thereby offering targeted benefits in topical applications.

## Data Availability

Not applicable.

## Abbreviations

The following abbreviations are used in this manuscript:

CD44	Cluster of differentiation 44
CEMIP	Cell migration-inducing hyaluronidase
FDA	Food drug administration
HA	Hyaluronic acid
HAS	Hyaluronan synthase
HMWHA	High-molecular-weight hyaluronic acid
HYAL	Hyaluronidase
ISO	International standard organization
LMWHA	Low-molecular-weight hyaluronic acid
LYVE1	Lymphatic vessel endothelial hyaluronan receptor-1
MMWHA	Medium molecular weight hyaluronic acid
P2X7	Purinergic receptor P2X7
RHAMM	Receptor for hyaluronan-mediated motility
SEM	Scanning electron microscopy
TLR	Toll-like receptor
TMEM2	Transmembrane protein 2
UV	Ultraviolet
